# Taking a Computational Cultural Neuroscience Approach to Study Parent-Child Similarities in Diverse Cultural Contexts

**DOI:** 10.3389/fnhum.2021.703999

**Published:** 2021-08-26

**Authors:** Pin-Hao A. Chen, Yang Qu

**Affiliations:** ^1^Department of Psychology, National Taiwan University, Taipei, Taiwan; ^2^Neurobiology and Cognitive Science Center, National Taiwan University, Taipei, Taiwan; ^3^Center for Artificial Intelligence and Advanced Robotics, National Taiwan University, Taipei, Taiwan; ^4^School of Education and Social Policy, Northwestern University, Evanston, IL, United States

**Keywords:** cultural neuroscience, developmental neuroscience, computational neuroscience, parent-child interaction, social interaction, neuroimaging

## Abstract

Parent-child similarities and discrepancies at multiple levels provide a window to understand the cultural transmission process. Although prior research has examined parent-child similarities at the belief, behavioral, and physiological levels across cultures, little is known about parent-child similarities at the neural level. The current review introduces an interdisciplinary computational cultural neuroscience approach, which utilizes computational methods to understand neural and psychological processes being involved during parent-child interactions at intra- and inter-personal level. This review provides three examples, including the application of intersubject representational similarity analysis to analyze naturalistic neuroimaging data, the usage of computer vision to capture non-verbal social signals during parent-child interactions, and unraveling the psychological complexities involved during real-time parent-child interactions based on their simultaneous recorded brain response patterns. We hope that this computational cultural neuroscience approach can provide researchers an alternative way to examine parent-child similarities and discrepancies across different cultural contexts and gain a better understanding of cultural transmission processes.

## Introduction

Cultural values, beliefs, and practices are transmitted across generations (Matsumoto and Yoo, 2006; Kitayama et al., 2019). Parents play a key role in this cultural socialization process, because they are not only knowledgeable about cultural values and norms in their societies, but also responsible for guiding children’s development and conveying such cultural values and norms to children. Through parenting practices and parent-child interactions, either explicitly or implicitly, children may adopt cultural values and norms, which further influence children’s psychological and behavioral adjustment (Grusec and Goodnow, 1994; Berry and Georgas, 2009; Bornstein, 2012). Therefore, it is critical to investigate parent-child similarities at different levels across cultures to fully understand the cultural socialization process. Indeed, past research has paid attention to parent-child similarities at the belief, behavioral, and physiological levels (Feldman, 2007; Papp et al., 2009; Chen et al., 2014; Kim et al., 2015). With the recent advances in cultural neuroscience, more research is needed to understand parent-child similarities at the neural level. The current review highlights a newly emerging computational cultural neuroscience approach, which can provide unique insights into parent-child similarities and discrepancies across cultures. Advances in this field have the potential to not only inform the understanding of cultural transmission by elucidating how children’s brains are wired to their parents in the dynamic process of cultural learning, but also identify differences in parent-child neural similarities either across cultures or within the same culture and examine predictors and consequences of such differences.

## Parent-Child Similarities and Discrepancies Across Cultures

Past research has examined parent-child similarities and discrepancies in beliefs, behavior, and physiological adjustment across diverse cultural contexts. Guided by the value transmission model (Grusec and Goodnow, 1994), parent-child similarities at the belief level provide an important window to understand the socialization and internalization of cultural values and norms. Cross-cultural research suggests that the extent to which children internalize and adopt their parents’ goals may differ across cultures. For example, intergenerational transmission of parental goals from mothers to children appeared to be weaker among Americans than Chinese (Qu et al., 2016). Moreover, much attention has been paid to minority families in the United States, focusing on parent-child similarities and discrepancies in cultural values and beliefs. Based on parents and children’s self-reported cultural orientations, prior studies have employed different approaches to quantify parent-child gaps (e.g., different score approach, interaction approach, or match/mismatch groups; for a review, see Birman, 2006). This line of research not only documents important parent-child gaps in cultural orientations, but also illustrates the impact of such gaps on children’s adjustment, such as academic achievement and socioemotional well-being (Chen et al., 2014; Kim et al., 2015). For example, Chinese American children tend to show greater externalizing problems among families in which children are low in Chinese proficiency while their parents are high in Chinese proficiency (Chen et al., 2014). Moreover, Chinese American adolescents who are more Chinese-oriented than their parents tend to have lower scores in English language arts (Kim et al., 2015).

Decades of research in developmental psychology has studied parent-child similarity or synchrony at the behavioral level (for a review, see Harrist and Waugh, 2002). In addition, studies have examined whether parent-child dyads show similarities in their perception of the same type of behavior (e.g., parenting practices) from a cross-cultural lens (Cheung et al., 2016). For example, a recent meta-analysis on parent-adolescent discordance in perceptions of parenting found that the levels of parent-adolescent discordance were higher in more individualistic societies, such as the United States than in other societies with lower levels of individualism (Hou et al., 2020). In the past decade, scholars also move beyond the belief and behavioral levels, and start investigating the similarity at the physiological level in parent-child dyads with diverse sociocultural backgrounds (Papp et al., 2009; Suveg et al., 2016; Han et al., 2019; Byrd-Craven et al., 2020).

## Taking a Computational Cultural Neuroscience Approach to Study Parent-Child Similarities

In this review, we focus on the investigation of parent-child similarities across diverse cultures at the neural level. To examine cultural differences in parent-child similarities in naturalistic settings, we propose an interdisciplinary approach called *Computational Cultural Neuroscience*, which utilizes computational methods to understand what neural and psychological processes are involved during interaction at both intrapersonal and interpersonal levels. In order to obtain measurements at both levels, this approach not only relies on the brain measurement, but also takes advantage of behavioral measurement, such as self-reports, task-based behavioral measurement, and real-time behavioral recording. In this review, we introduce recent development in computational methods which could be specifically applied to study parent-child similarities across diverse cultural contexts, including using intersubject representational similarity analysis to analyze naturalistic neuroimaging data, utilizing computer vision to analyze non-verbal social interactions, and employing pre-trained brain signatures to analyze hyperscanning data.

### Naturalistic Neuroimaging and Intersubject Representational Similarity Analysis

The extent to which parents and children are similar to each other is shaped, in part, by their culture. Thus, researchers can examine whether parents or children with the same cultural background think or feel in a more similar way than those with different cultural backgrounds and how these differences in thoughts and feelings are encoded in the brain (Chen et al., 2020). In order to elicit real-world thoughts and feelings, complex naturalistic real-world stimuli are needed (Cantlon and Li, 2013), and participants are asked to watch or listen to exactly the same stimuli. Naturalistic neuroimaging, a recent growing field in neuroimaging, aims to use naturalistic stimuli to elicit complex experiences closer to what individuals experience in daily life (Hasson et al., 2008; Vanderwal et al., 2017; Nastase et al., 2019, 2020; Chen et al., 2020; Finn and Bandettini, 2020). In the past decade, this approach has been used to explore various topics, such as shared memory (Baldassano et al., 2017; Chen et al., 2017), memory transmission (Zadbood et al., 2017), speaker-listener communication (Stephens et al., 2010), and narrative comprehension (Simony et al., 2016; Yeshurun et al., 2017; Nguyen et al., 2019). In two recent studies, researchers found that political identities shape neural synchrony when individuals watch the same stimuli, such that those with the same identity showed highly synchronized neural responses to the same naturalistic political contents (Leong et al., 2020; van Baar et al., 2021). In line with the above finding, researchers also found that for individuals with similar personality traits (Finn et al., 2018, 2020; Chen et al., 2020; Finn and Bandettini, 2020) or with closer social distances from the same social network (Parkinson et al., 2017, 2018), they tend to show similar neural responses to the same naturalistic content. This suggests that differences in personality, identity or cultural background may contribute to differences in neural processes of information.

Combining with the naturalistic neuroimaging approach, intersubject correlation (ISC), a commonly used method in neuroimaging, has enabled researchers to explore shared responses across different brains (Nastase et al., 2019; Chen et al., 2020). For example, Stephens et al. (2010) tested how shared information between listeners and speakers are represented in the brain during communication. A similar method can be applied to examine whether parents and children from the same cultural background also show similar neural fluctuations to the naturalistic viewing paradigm, especially when the viewing content is related to parenting, family, and education. By calculating the pairwise correlation across all possible pairs, the similarity of brain responses within the same cultural group and the dissimilarity of the responses between different cultural groups would be revealed. Although the above analysis can identify the shared neural dynamics across individuals within the same cultural group, individual variations in attitudes or cultural beliefs might also exist within the same group.

**FIGURE 1:**
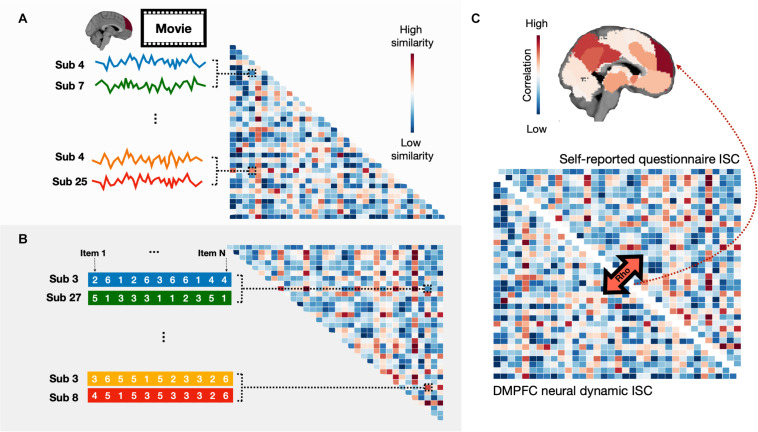
Intersubject correlation (ISC) and intersubject representational similarity analysis (IS-RSA). IS-RSA involves **(A)** computing an ISC, which yields a subject by subject correlation matrix using mean neural responses within an ROI (e.g., DMPFC) for a movie, **(B)** computing another ISC, which yields a subject by subject correlation matrix using item-wise responses for self-reported questionnaires (e.g., parenting beliefs), and **(C)** conducting a spearman rank correlation between the two correlation matrices. In this case, intersubject variability in the DMPFC is well captured by intersubject variability in parenting beliefs.

Intersubject representational similarity analysis (IS-RSA), a newly developed computational method in neuroimaging, is implemented to search the mapping between individual variations in experiences during naturalistic viewing and variations in preferences or other differences (Chen et al., 2020; Finn et al., 2020). For example, Chen et al. (2020) found that individuals with similar preferences in sociosexual desire also exhibited similar neural dynamics in the brain default mode, mentalizing, and reward networks when watching erotic movies. By contrast, individuals with similar preferences in self-control showed similar neural responses in the brain fronto-parietal executive control network. Thus, future studies could use IS-RSA to explore whether intersubject similarities in parenting beliefs are related to their similarities in neural dynamics involved in parenting experiences. For example, researchers could recruit a group of parents to watch a series of parenting-related movies in the fMRI scanner, aiming to elicit their parenting-related experiences without interrupting the formation of these experiences. Then, ISC is used to compute a subject by subject correlation matrix using mean neural responses within an ROI (e.g., DMPFC) during the movie watching part (Figure 1A). After scanning, researchers could ask these participants to complete questionnaires related to their parenting beliefs or even to collect measurements of their parenting practices in daily life, and ISC is used to compute a subject by subject correlation matrix using item-wise responses for self-reported questionnaires (Figure 1B). Lastly, IS-RSA is used to find the correspondence between individual variations in parenting beliefs or behaviors and variations in neural dynamics, representing their experiences during naturalistic neuroimaging (Figure 1C). The same method can then be used to examine whether parents and children with the same cultural background think or feel in a more similar way than those with different cultural backgrounds as well as how these differences in thoughts and feelings are represented in the brain. By taking this approach, developmental and cultural neuroscientists could expand their previous findings by using RSA (Lee et al., 2017, 2018) to discover more complex patterns of intersubject (i.e., parent and children) similarities in different cultural groups. Therefore, ISC and IS-RSA could eventually open another exciting venue for future cultural psychologists to explore cross-cultural differences, especially in parenting and education.

### Analyzing Non-verbal Social Signals During Real-Time Communication

Social communications between parents and children not only rely on verbal content, but also depend on non-verbal behavior because some social signals contain cultural-specific meanings and are only expressed through non-verbal communications (Tsai, 2007; Jack and Schyns, 2015; Tsai et al., 2019). Thus, at the interpersonal level, examining non-verbal behavior, such as facial expressions or gestures during communications between parents and children in their dynamic real-time synchrony is a key to a deeper understanding of cultural differences in parent-child similarities, especially in their social communication (Jack and Schyns, 2015, 2017). Facial action coding system (FACS) is among one of the most popular coding systems to analyze human facial expressions (Ekman and Friesen, 1976). In the past, in order to accurately code facial expressions, researchers needed to spend hundreds of training hours to become a certified FACS coder. However, with recent rapid developments in artificial intelligence, machine learning and computer vision have enabled psychologists easier than before to decipher the myth of non-verbal behavior, such as subtle changes in facial expressions during real-time social interactions. For example, researchers have used pre-trained convolutional neural networks (CNN) to extract facial action units, a standardized system to describe the intensity of facial muscle movements (Littlewort et al., 2011; Senechal et al., 2012; McDuff et al., 2013). In recent years, open source softwares, such as Openface 2.0 (Baltrusaitis et al., 2018) or Py-Feat (Python Facial Expression Analysis Toolbox; Cheong et al., 2021) have made their pre-trained CNN available for researchers around the world to use their tools for free. Today, researchers who are interested in analyzing interpersonal influence on facial expressions have a fast and reliable way to get a deeper understanding of the functions of these non-verbal communications than ever before.

To understand the cultural differences in parent-child similarities, researchers could ask whether specific facial behavioral patterns may be shown during parent-child interaction in some cultural groups, and whether these behavioral patterns signal any particular psychological meaning. For example, previous cross-cultural facial expression research found that, although some expressions (e.g., pain) were similarly represented, some expressions (e.g., smiling) were distinctly represented across different cultures, suggesting that the communication purpose of expressions might differ across cultures (Chen and Jack, 2017; Chen et al., 2018; Chen C. et al., 2019; Tsai et al., 2019). This real-time communication study can further be combined with a naturalistic viewing paradigm, in which experimenters record how a parent-child dyad responds to the same naturalistic stimuli and further influence their conjunctional impressions about the same stimuli. For example, a recent study combined a naturalistic viewing paradigm with facial behavioral recording to examine whether two individuals show more facial expression synchrony when they watch a TV show together in the same room than separately in a different room, and whether this synchrony carries any psychological meaning (Cheong et al., 2020). They found that participants revealed more facial synchrony not only in temporal dynamics, but also in spatial patterns when they watched the show together. Most importantly, higher facial synchrony predicted higher feelings of social connection with the dyad who watched the show together, suggesting that the facial expression synchrony is measurable and has its predictive value in dyadic interaction. In another study, researchers took machine-learning tools to extract distinct features of action units and established a facial expression model of pain experience. They further used this model to predict whether the communication of beliefs from doctors to patients impacted patients’ pain experience (Chen P.-H. A. et al., 2019). They found that beliefs of treatment effectiveness from doctors were revealed on their facial expressions, and patients may acquire doctors’ beliefs during social interaction. These two examples demonstrate that analyzing facial expressions by machine-learning tools could help researchers to examine not only what kinds of social signals occur during dyadic interactions, but also the impact of these signals on the quality of relationships. Thus, future studies could recruit parent-child dyads with different cultural backgrounds and record their facial expression as well as gestures during their real-time social interactions. By using pre-trained CNN models, researchers could analyze whether any cultural-specific facial expressions between parents and children could predict their communication outcomes or relationship satisfaction. Using computational methods to study parent-child interaction could help researchers to explore cultural-specific variations in dynamic parent-child interactions and to provide a more comprehensive understanding of the cultural socialization process across developmental stages.

### The Usage of Brain Signatures in Analyzing Hyperscanning Data

Although analyzing non-verbal behaviors during dyadic interaction could help researchers to examine what kinds of social signals might occur during interaction, it is still difficult to disentangle what kinds of psychological processes might occur within each individual during their interactions. Thus, in this section, we suggest that researchers could take a hybrid approach to decode the complexity of psychological processes during dyadic interactions by collecting dyadic brain data with hyperscanning techniques, and analyzing these data with brain signatures established via computational methods (Woo et al., 2017; Gerloff et al., 2021). By taking this new approach, researchers can further the understanding of parent-child similarities across diverse cultural contexts.

Hyperscanning aims to measure brain responses for the two individuals simultaneously during their social interactions. Hyperscanning has been used to examine diverse research topics, including dyadic trust decision-making (Krueger et al., 2007), social interactions between teachers and students (Nguyen et al., 2020), cooperative behaviors between parents and children (Reindl et al., 2018; Miller et al., 2019), infant-adult interaction during natural communication (Piazza et al., 2020), and even doctor-patient interaction during their acupuncture treatment (Ellingsen et al., 2020). Conventional experimental designs in neuroimaging, which measures brain responses in a single individual one at a time, make researchers difficult to explore what psychological processes might be involved during real-time dyadic interactions. Compared to conventional designs, hyperscanning enables researchers to collect dynamic brain responses of interacting individuals during their real-time interactions, which reflects the true psychological complexities from dyadic interactions.

Since multiple psychological processes might be simultaneously involved during dyadic interactions, brain signatures established via computational methods are particularly useful to decode what psychological processes might occur during parent-child interaction (Woo et al., 2017; Kragel et al., 2018). In general, the first step in developing a brain signature is to use machine-learning algorithms to establish brain patterns to accurately predict outcomes (e.g., making a trust or distrust decision) in a training dataset (Chen et al., 2021). Unlike the conventional inferential statistical method using all data points to make inference, the first step of developing a brain signature usually involves a cross-validation procedure within the training dataset. For example, this procedure begins with splitting the training dataset into N folds (e.g., five folds) (Chang et al., 2015). A machine-learning algorithm (e.g., support vector machine) is applied to train a brain signature in fourfolds of the data, and test the performance (e.g., accuracy) of this signature in the remaining onefold. Since each fold could be the testing data during this cross-validation step, researchers then iterate this procedure until each of the fivefolds of the data has been tested, and an averaged accuracy across these five testing folds is calculated to represent the performance of this brain signature. Although the performance of a brain signature can be evaluated through this cross-validation procedure, it is only the first step in establishing a brain signature. Most importantly, the second step is applying this brain signature to make an out-of-sample prediction. The evaluated criteria is to test whether a similar predictive performance of this brain signature could be found in a hold-out dataset that has not been used in the first step. This second step is crucial in testing whether the predictivity and generalizability of this brain signature can be validated across datasets.

The above approach has been successfully used in predicting not only basic sensations, such as pain intensity (Wager et al., 2013) or sustained clinical pain (Lee et al., 2021), but also diverse kinds of feelings, such as vicarious pain (Krishnan et al., 2016), picture-induced negative affect (Chang et al., 2015), feelings of guilt (Yu et al., 2020), and even empathetic care and distress (Ashar et al., 2017). These examples demonstrate that, with the growing popularity in data sharing, neuroscientists would be able to establish brain models representing different psychological processes. By collecting parent-child interaction brain data via hyperscanning techniques, researchers can use these pre-trained brain signatures to examine what psychological processes might occur within interacting individuals and when these processes occur over the course of their interaction. This combined approach would help developmental and cultural neuroscientists well-equipped to unravel the complexities of psychological processes and to further explore parent-child similarities or discrepancies across diverse cultural contexts.

## Contributions of Computational Cultural Neuroscience to the Field

Computational cultural neuroscience approach provides powerful tools that can make important and unique contributions to the field. First, this approach can inform the understanding of cultural transmission, providing insights into how cultural values and practices are passed on across generations not only at belief and behavioral level, but also at the neural level. For example, researchers can examine cultural similarities and differences in neural synchrony within parent-child dyads when they engage in interactions or communications. As parenting practices or behaviors are shaped, in part, by their culture, computational cultural neuroscience provides techniques to capture how children’s brains are wired to their parents in the dynamic process of cultural learning.

Second, in addition to examining between-culture variability in parent-child dyads at the neural level, this approach allows researchers to explore individual differences across families within the same culture, which can elucidate the cultural transmission process at the individual level. Within each culture, there is likely to be variability in the extent to which parents adopt cultural values and norms, thereby creating variability in parent-child interactions and ultimately children’s development. Therefore, parent-child neural similarities may represent meaningful differences across families within the same culture, and it is important to examine what factors contribute to such differences. For example, using representational similarity analysis, recent research suggests that on average, American mothers and their children do not show similar neural patterns during stress (Lee et al., 2018). However, parent-child neural similarities vary based on different levels of family connectedness, such that only dyads reporting high family connectedness show similar neural profiles. Moreover, greater neural similarity is associated with reduced stress in adolescents, suggesting that shared neural profiles in parent-child dyads enhance adolescents’ psychological well-being. In addition, this computational cultural neuroscience approach provides a new window to study parent-child gaps in acculturation processes (Chen et al., 2013, 2015a,b, 2015c). Moving beyond prior studies on differences in parent-child beliefs (e.g., cultural orientations) and behavior, researchers can examine different neural patterns in parent-child dyads across different immigrant families as they make decisions, to better understand intergenerational conflict in immigrant families.

Third, this approach will also shed light on the links between beliefs, brain, and behavior. For example, it is informative to investigate how belief similarities in parent-child dyads are related to brain similarities, and how brain similarities in parent-child dyads are linked to behavioral similarities. More importantly, future research can explore the meaning and developmental consequences when parent-child similarity at one level does not match with the similarity at another level. This line of research will not only extend prior research that links beliefs, brain, and behavior at the intrapersonal level (i.e., within the same person), but also explore the complex relations among beliefs, brain, and behavior at the interpersonal level (i.e., in parent-child dyad).

Although this computational cultural neuroscience approach provides a novel perspective to examine parent-child similarities in diverse cultural contexts, limitations of this approach should be noted. First, although using pre-trained brain signatures to analyze hyperscanning data can help researchers to examine what psychological processes might occur within interacting individuals, it is still possible that some important psychological processes are not covered by pre-trained brain signatures. Thus, more future studies are needed to establish brain signatures representing most relevant psychological processes during social interactions. Second, most of the methods described in this review are designed for cross-sectional studies, but meaningful cultural changes in attitudes or behavior can provide important information in longitudinal studies. For instance, a previous longitudinal study showed that even in the same cohort of immigrants, changes in self-construal during the process of acculturation were reflected in the relative engagement of brain areas implicated in self-referential processing (Chen et al., 2015b). Thus, future studies could combine this computational cultural neuroscience approach with a longitudinal research design to examine how neural representations are dynamically influenced by immigrants’ acculturated cultural norms during their acculturation processes, and whether their changes during acculturation influence their parent-child interactions.

## Conclusion

Taken together, this integrative review highlights the importance of understanding parent-child similarities and discrepancies at the neural level across cultures. We also introduce several cutting-edge computational methods to explore intersubject neural similarities, decode social signals during communications, and unravel the complexities of real-time parent-child interactions based on their simultaneous recorded brain response patterns. This computational cultural neuroscience approach aims to promote the use of computational methods to get a deeper understanding of cultural transmission processes, and hope to provide theoretical accounts and innovative tools for this promising line of research.

## Author Contributions

Both authors listed have made a substantial, direct and intellectual contribution to the work, and approved it for publication.

## Conflict of Interest

The authors declare that the research was conducted in the absence of any commercial or financial relationships that could be construed as a potential conflict of interest.

## Publisher’s Note

All claims expressed in this article are solely those of the authors and do not necessarily represent those of their affiliated organizations, or those of the publisher, the editors and the reviewers. Any product that may be evaluated in this article, or claim that may be made by its manufacturer, is not guaranteed or endorsed by the publisher.

## References

[B1] Ashar Y. K. Andrews-Hanna J. R. Dimidjian S. Wager T. D. (2017). Empathic care and distress: predictive brain markers and dissociable brain systems. *Neuron* 94 1263–1273.e. 28602689 10.1016/j.neuron.2017.05.014 PMC5532453

[B2] Baldassano C. Chen J. Zadbood A. Pillow J. W. Hasson U. Norman K. A. (2017). Discovering event structure in continuous narrative perception and memory. *Neuron* 95 709–721.e. 28772125 10.1016/j.neuron.2017.06.041 PMC5558154

[B3] Baltrusaitis T. Zadeh A. Lim Y. C. Morency L. (2018). “OpenFace 2.0: facial behavior analysis toolkit,” in *Proceedings of the 2018 13th IEEE International Conference on Automatic Face Gesture Recognition (FG 2018)* (Piscataway, NJ: IEEE), 59–66.

[B4] Berry J. W. Georgas J. (2009). “An ecocultural perspective on cultural transmission: the family across cultures,” in *Cultural Transmission: Psychological, Developmental, Social, and Methodological Aspects*, ed. Schönpflug U. (Cambridge: Cambridge University Press), 95–125. 10.1017/cbo9780511804670.007

[B5] Birman D. (2006). “Measurement of the acculturation gap in immigrant families and implications for parent–child relationships,” in *Acculturation and Parent-Child Relationships*, ed. Bornstein M. Cotes L. (Mahwah, NJ: LEA),113–134. 10.4324/9780415963589-7

[B6] Bornstein M. H. (2012). Cultural approaches to parenting. *Parent. Sci. Pract.* 12 212–221. 10.1080/15295192.2012.683359 22962544 PMC3433059

[B7] Byrd-Craven J. Criss M. M. Calvi J. L. Cui L. Baraldi A. Sheffield Morris A. (2020). Adrenocortical attunement, reactivity, and potential genetic correlates among parent-daughter dyads from low-income families. *Dev. Psychobiol.* 62 1035–1045. 10.1002/dev.21970 32291754 PMC7554072

[B8] Cantlon J. F. Li R. (2013). Neural activity during natural viewing of Sesame Street statistically predicts test scores in early childhood. *PLoS Biol.* 11:e1001462. 10.1371/journal.pbio.1001462 23300385 PMC3536813

[B9] Chang L. J. Gianaros P. J. Manuck S. B. Krishnan A. Wager T. D. (2015). A sensitive and specific neural signature for picture-induced negative affect. *PLoS Biol.* 13:e1002180. 10.1371/journal.pbio.1002180 26098873 PMC4476709

[B10] Chen C. Jack R. E. (2017). Discovering cultural differences (and similarities) in facial expressions of emotion. *Curr. Opin. Psychol.* 17 61–66. 10.1016/j.copsyc.2017.06.010 28950974

[B11] Chen C. Crivelli C. Garrod O. G. B. Schyns P. G. Fernández-Dols J.-M. Jack R. E. (2018). Distinct facial expressions represent pain and pleasure across cultures. *Proc. Natl. Acad. Sci. U.S.A.* 115 E10013–E10021. 30297420 10.1073/pnas.1807862115 PMC6205428

[B12] Chen C. Hensel L. B. Duan Y. Ince R. A. A. Garrod O. G. B. Beskow J. (2019). “Equipping social robots with culturally-sensitive facial expressions of emotion using data-driven methods,” in *Proceedings of the 2019 14th IEEE International Conference on Automatic Face Gesture Recognition (FG 2019)* (Piscataway, NJ: IEEE), 1–8.

[B13] Chen J. Leong Y. C. Honey C. J. Yong C. H. Norman K. A. Hasson U. (2017). Shared memories reveal shared structure in neural activity across individuals. *Nat. Neurosci.* 20 115–125. 10.1038/nn.4450 27918531 PMC5191958

[B14] Chen P.-H. A. Cheong J. H. Jolly E. Elhence H. Wager T. D. Chang L. J. (2019). Socially transmitted placebo effects. *Nat. Hum. Behav.* 3 1295–1305. 10.1038/s41562-019-0749-5 31636406 PMC7494051

[B15] Chen P.-H. A. Fareri D. Güroğlu B. Delgado M. R. Chang L. J. (2021). towards a neurometric-based construct validity of trust. *bioRxiv* [Preprint]. 10.1101/2021.07.04.451074

[B16] Chen P.-H. A. Heatherton T. F. Freeman J. B. (2015a). “Brain-as-predictor approach: an alternative way to explore acculturation processes,” in *Neuroscience in Intercultural Contexts*, Vol. 1 eds Warnick J. E. Landis D. (New York, NY: Springer), 143–170. 10.1007/978-1-4939-2260-4_6

[B17] Chen P.-H. A. Jolly E. Cheong J. H. Chang L. J. (2020). Intersubject representational similarity analysis reveals individual variations in affective experience when watching erotic movies. *Neuroimage* 216:116851. 10.1016/j.neuroimage.2020.116851 32294538 PMC7955800

[B18] Chen P.-H. A. Wagner D. D. Kelley W. M. Heatherton T. F. (2015b). Activity in cortical midline structures is modulated by self-construal changes during acculturation. *Cult. Brain* 3 39–52. 10.1007/s40167-015-0026-z 26236572 PMC4520324

[B19] Chen P.-H. A. Wagner D. D. Kelley W. M. Powers K. E. Heatherton T. F. (2013). Medial prefrontal cortex differentiates self from mother in Chinese: evidence from self-motivated immigrants. *Cult. Brain* 1 3–15. 10.1007/s40167-013-0001-5

[B20] Chen P.-H. A. Whalen P. J. Freeman J. B. Taylor J. M. Heatherton T. F. (2015c). Brain reward activity to masked in-group smiling faces predicts friendship development. *Soc. Psychol. Personal. Sci.* 6 415–421. 10.1177/1948550614566093 26185595 PMC4501035

[B21] Chen S. H. Hua M. Zhou Q. Tao A. Lee E. H. Ly J. (2014). Parent–child cultural orientations and child adjustment in Chinese American immigrant families. *Dev. Psychol.* 50 189–201. 10.1037/a0032473 23566081

[B22] Cheong J. H. Molani Z. Sadhukha S. Chang L. J. (2020). Synchronized affect in shared experiences strengthens social connection. *arXiv* [Preprint]. 10.31234/osf.io/bd9wn PMC10613250 37898664

[B23] Cheong J. H. Xie T. Byrne S. Chang L. J. (2021). Py-Feat: python facial expression analysis toolbox. *arXiv* [Preprint]. arXiv [cs.CV] 10.1007/s42761-023-00191-4 PMC10751270 38156250

[B24] Cheung C. S. Pomerantz E. M. Wang M. Qu Y. (2016). Controlling and autonomy-supportive parenting in the United States and China: beyond children’s reports. *Child Dev.* 87 1992–2007. 10.1111/cdev.12567 27317628

[B25] Ekman P. Friesen W. V. (1976). Measuring facial movement. *Environ. Psychol. Nonverbal Behav.* 1 56–75. 10.1007/bf01115465

[B26] Ellingsen D.-M. Isenburg K. Jung C. Lee J. Gerber J. Mawla I. (2020). Dynamic brain-to-brain concordance and behavioral mirroring as a mechanism of the patient-clinician interaction. *Sci. Adv.* 6:eabc1304. 10.1126/sciadv.abc1304 33087365 PMC7577722

[B27] Feldman R. (2007). Parent-infant synchrony and the construction of shared timing; physiological precursors, developmental outcomes, and risk conditions. *J. Child Psychol. Psychiatry* 48 329–354. 10.1111/j.1469-7610.2006.01701.x 17355401

[B28] Finn E. S. Bandettini P. A. (2020). Movie-watching outperforms rest for functional connectivity-based prediction of behavior. *bioRxiv* [Preprint]. 10.1101/2020.08.23.263723 PMC8204673 33813007

[B29] Finn E. S. Corlett P. R. Chen G. Bandettini P. A. Constable R. T. (2018). Trait paranoia shapes inter-subject synchrony in brain activity during an ambiguous social narrative. *Nat. Commun.* 9:2043. 10.1038/s41467-018-04387-2 PMC5966466 29795116

[B30] Finn E. S. Glerean E. Khojandi A. Y. Nielson D. Molfese P. J. Handwerker D. A. (2020). Idiosynchrony: from shared responses to individual differences during naturalistic neuroimaging. *Neuroimage* 215:116828. 10.1016/j.neuroimage.2020.116828 32276065 PMC7298885

[B31] Gerloff C. Konrad K. Bzdok D. Büsing C. Reindl V. (2021). Interacting brains revisited: a cross-brain network neuroscience perspective. *bioRxiv* [Preprint]. 10.1002/hbm.25966 PMC9435014 35661477

[B32] Grusec J. E. Goodnow J. J. (1994). Impact of parental discipline methods on the child’s internalization of values: a reconceptualization of current points of view. *Dev. Psychol.* 30:4. 10.1037/0012-1649.30.1.4

[B33] Han Z. R. Gao M. M. Yan J. Hu X. Zhou W. Li X. (2019). Correlates of parent-child physiological synchrony and emotional parenting: differential associations in varying interactive contexts. *J. Child Family Studies* 28 1116–1123. 10.1007/s10826-019-01337-4

[B34] Harrist A. W. Waugh R. M. (2002). Dyadic synchrony: its structure and function in children’s development. *Dev. Rev.* 22 555–592. 10.1016/s0273-2297(02)00500-2

[B35] Hasson U. Landesman O. Knappmeyer B. Vallines I. Rubin N. Heeger D. J. (2008). Neurocinematics: the neuroscience of film. *Projections* 2 1–26. 10.3167/proj.2008.020102

[B36] Hou Y. Benner A. D. Kim S. Y. Chen S. Spitz S. Shi Y. (2020). Discordance in parents’ and adolescents’ reports of parenting: a meta-analysis and qualitative review. *Am. Psychol.* 75 329–348. 10.1037/amp0000463 31192619 PMC10624508

[B37] Jack R. E. Schyns P. G. (2015). The human face as a dynamic tool for social communication. *Curr. Biol.* 25 R621–R634. 26196493 10.1016/j.cub.2015.05.052

[B38] Jack R. E. Schyns P. G. (2017). Toward a social psychophysics of face communication. *Ann. Rev. Psychol.* 68 269–297. 10.1146/annurev-psych-010416-044242 28051933

[B39] Kim S. Y. Wang Y. Chen Q. Shen Y. Hou Y. (2015). Parent-child acculturation profiles as predictors of Chinese American adolescents’ academic trajectories. *J. Youth Adolesc.* 44 1263–1274. 10.1007/s10964-014-0131-x 24820295 PMC4231017

[B40] Kitayama S. Varnum M. E. W. Salvador C. E. (2019). “Cultural neuroscience,” in *Handbook of Cultural Psychology*, 2nd Edn, eds Cohen D. Kitayama S. (New York, NY: The Guilford Press), 79–118.

[B41] Kragel P. A. Koban L. Barrett L. F. Wager T. D. (2018). Representation, pattern information, and brain signatures: from neurons to neuroimaging. *Neuron* 99 257–273. 10.1016/j.neuron.2018.06.009 30048614 PMC6296466

[B42] Krishnan A. Woo C.-W. Chang L. J. Ruzic L. Gu X. Lopez-Sola M. (2016). Somatic and vicarious pain are represented by dissociable multivariate brain patterns. *ELife* 5:e15166. 10.7554/eLife.15166 27296895 PMC4907690

[B43] Krueger F. McCabe K. Moll J. Kriegeskorte N. Zahn R. Strenziok M. (2007). Neural correlates of trust. *Proc. Natl. Acad. Sci.U.S.A.* 104 20084–20089. 18056800 10.1073/pnas.0710103104 PMC2148426

[B44] Lee J.-J. Kim H. J. Čeko M. Park B.-Y. Lee S. A. Park H. (2021). A neuroimaging biomarker for sustained experimental and clinical pain. *Nat. Med.* 27 174–182. 10.1038/s41591-020-1142-7 33398159 PMC8447264

[B45] Lee T.-H. Qu Y. Telzer E. H. (2017). Love flows downstream: mothers’ and children’s neural representation similarity in perceiving distress of self and family. *Soc. Cogn. Affect. Neurosci.* 12 1916–1927. 10.1093/scan/nsx125 29069521 PMC5716095

[B46] Lee T.-H. Qu Y. Telzer E. H. (2018). Dyadic neural similarity during stress in mother-child dyads. *J. Res. Adolesc.* 28 121–133. 10.1111/jora.12334 29460351 PMC6402773

[B47] Leong Y. C. Chen J. Willer R. Zaki J. (2020). Conservative and liberal attitudes drive polarized neural responses to political content. *Proc. Natl. Acad. Sci. U.S.A.* 117 27731–27739. 10.1073/pnas.2008530117 33082227 PMC7959490

[B48] Littlewort G. Whitehill J. Wu T. Fasel I. Frank M. Movellan J. (2011). The computer expression recognition toolbox (CERT). *Face Gesture* 2011 298–305.

[B49] Matsumoto D. Yoo S. H. (2006). Toward a new generation of cross-cultural research. *Perspect. Psychol. Sci.* 1 234–250. 10.1111/j.1745-6916.2006.00014.x 26151631

[B50] McDuff D. Kaliouby R. Senechal T. Amr M. Cohn J. Picard R. (2013). “Affectiva-mit facial expression dataset (am-fed): Naturalistic and spontaneous facial expressions collected,” in *Proceedings of the IEEE Conference on Computer Vision and Pattern Recognition Workshops* (Piscataway, NJ: IEEE), 881–888.

[B51] Miller J. G. Vrtička P. Cui X. Shrestha S. Hosseini S. M. H. Baker J. M. (2019). Inter-brain synchrony in mother-child dyads during cooperation: an fNIRS hyperscanning study. *Neuropsychologia* 124 117–124. 10.1016/j.neuropsychologia.2018.12.021 30594570 PMC6937429

[B52] Nastase S. A. Gazzola V. Hasson U. Keysers C. (2019). Measuring shared responses across subjects using intersubject correlation. *Soc. Cogn. Affect. Neurosci.* 14 667–685. 31099394 10.1093/scan/nsz037 PMC6688448

[B53] Nastase S. A. Goldstein A. Hasson U. (2020). Keep it real: rethinking the primacy of experimental control in cognitive neuroscience. *Neuroimage* 222:117254. 10.1016/j.neuroimage.2020.117254 32800992 PMC7789034

[B54] Nguyen M. Chang A. Micciche E. Meshulam M. (2020). Teacher-student neural coupling during teaching and learning. *bioRxiv* [Preprint]. 10.1101/2020.05.07.082958v1.abstract PMC8972247 34450637

[B55] Nguyen M. Vanderwal T. Hasson U. (2019). Shared understanding of narratives is correlated with shared neural responses. *Neuroimage* 184 161–170. 10.1016/j.neuroimage.2018.09.010 30217543 PMC6287615

[B56] Papp L. M. Pendry P. Adam E. K. (2009). Mother-adolescent physiological synchrony in naturalistic settings: within-family cortisol associations and moderators. *J. Fam. Psychol.* 23 882–894. 10.1037/a0017147 20001147 PMC2819131

[B57] Parkinson C. Kleinbaum A. M. Wheatley T. (2017). Spontaneous neural encoding of social network position. *Nat. Hum. Behav.* 1:0072.

[B58] Parkinson C. Kleinbaum A. M. Wheatley T. (2018). Similar neural responses predict friendship. *Nat. Commun.* 9:332. 10.1038/s41467-017-02722-7 PMC5790806 29382820

[B59] Piazza E. A. Hasenfratz L. Hasson U. Lew-Williams C. (2020). Infant and adult brains are coupled to the dynamics of natural communication. *Psychol. Sci.* 31 6–17. 10.1177/0956797619878698 31845827 PMC6966249

[B60] Qu Y. Pomerantz E. M. Deng C. (2016). Mothers’ goals for adolescents in the United States and China: content and transmission. *J. Res. Adolesc.* 26 126–141. 10.1111/jora.12176 27019420 PMC4803081

[B61] Reindl V. Gerloff C. Scharke W. Konrad K. (2018). Brain-to-brain synchrony in parent-child dyads and the relationship with emotion regulation revealed by fNIRS-based hyperscanning. *Neuroimage* 178 493–502. 10.1016/j.neuroimage.2018.05.060 29807152

[B62] Senechal T. Rapp V. Salam H. Seguier R. Bailly K. Prevost L. (2012). Facial Action Recognition Combining Heterogeneous Features via Multikernel Learning. *IEEE Trans. Syst. Man Cybern. B Cybern.* 42 993–1005. 10.1109/tsmcb.2012.2193567 22623430

[B63] Simony E. Honey C. J. Chen J. Lositsky O. Yeshurun Y. Wiesel A. (2016). Dynamic reconfiguration of the default mode network during narrative comprehension. *Nat. Commun.* 7:12141. 10.1038/ncomms12141 PMC4960303 27424918

[B64] Stephens G. J. Silbert L. J. Hasson U. (2010). Speaker-listener neural coupling underlies successful communication. *Proc. Natl. Acad. Sci. U.S.A.* 107 14425–14430. 10.1073/pnas.1008662107 20660768 PMC2922522

[B65] Suveg C. Shaffer A. Davis M. (2016). Family stress moderates relations between physiological and behavioral synchrony and child self-regulation in mother-preschooler dyads. *Dev. Psychobiol.* 58 83–97. 10.1002/dev.21358 26376933 PMC4928691

[B66] Tsai J. L. (2007). Ideal affect: cultural causes and behavioral consequences. *Perspect. Psychol. Sci.* 2 242–259. 10.1111/j.1745-6916.2007.00043.x 26151968

[B67] Tsai J. L. Blevins E. Bencharit L. Z. Chim L. Fung H. H. Yeung D. Y. (2019). Cultural variation in social judgments of smiles: the role of ideal affect. *J. Pers. Soc. Psychol.* 116 966–988. 10.1037/pspp0000192 29902026

[B68] van Baar J. M. Halpern D. J. FeldmanHall O. (2021). Intolerance of uncertainty modulates brain-to-brain synchrony during politically polarized perception. *Proc. Natl. Acad. Sci. U.S.A.* 118:e2022491118. 10.1073/pnas.2022491118 33986114 PMC8157931

[B69] Vanderwal T. Eilbott J. Finn E. S. Craddock R. C. Turnbull A. Castellanos F. X. (2017). Individual differences in functional connectivity during naturalistic viewing conditions. *Neuroimage* 157 521–530. 10.1016/j.neuroimage.2017.06.027 28625875

[B70] Wager T. D. Atlas L. Y. Lindquist M. A. Roy M. Woo C.-W. Kross E. (2013). An fMRI-based neurologic signature of physical pain. *Engl. J. Med.* 368 1388–1397. 10.1056/nejmoa1204471 23574118 PMC3691100

[B71] Woo C.-W. Chang L. J. Lindquist M. A. Wager T. D. (2017). Building better biomarkers: brain models in translational neuroimaging. *Nat. Neurosci.* 20 365–377. 10.1038/nn.4478 28230847 PMC5988350

[B72] Yeshurun Y. Swanson S. Simony E. Chen J. Lazaridi C. Honey C. J. (2017). Same story. different story: the neural representation of interpretive frameworks. *Psychol. Sci.* 28 307–319. 10.1177/0956797616682029 28099068 PMC5348256

[B73] Yu H. Koban L. Chang L. J. Wagner U. Krishnan A. Vuilleumier P. (2020). A generalizable multivariate brain pattern for interpersonal guilt. *Cereb. Cortex* 6 3558–3572. 10.1093/cercor/bhz326 32083647 PMC7232998

[B74] Zadbood A. Chen J. Leong Y. C. Norman K. A. Hasson U. (2017). How we transmit memories to other brains: constructing shared neural representations via communication. *Cereb. Cortex* 27 4988–5000. 10.1093/cercor/bhx202 28922834 PMC6057550

